# Functional characterization of human iPSC–derived sensory neuron processes in microfluidic cultures

**DOI:** 10.1093/stcltm/szag065

**Published:** 2026-08-08

**Authors:** Laura Kleckner, Omar Mossad, Anil Kumar Kalia, Ramin Raouf

**Affiliations:** Wolfson Sensory, Pain and Regeneration Centre (SPaRC), Institute of Psychiatry, Psychology & Neuroscience, King’s College London, London, SE1 1UL, United Kingdom; Research and Development, Grünenthal GmbH, 52078 Aachen, Germany; Research and Development, Grünenthal GmbH, 52078 Aachen, Germany; Wolfson Sensory, Pain and Regeneration Centre (SPaRC), Institute of Psychiatry, Psychology & Neuroscience, King’s College London, London, SE1 1UL, United Kingdom

**Keywords:** iPSC-derived sensory neurons, microfluidic culture, compartmentalization, signal propagation, Nav1.7, Nav1.8

## Abstract

Rodent cell culture models have long underpinned research into nociceptive signaling; however, their limited recapitulation of human nociceptor biology has created a translational gap in analgesic development. While primary human sensory neurons are relevant, their use is hampered by scarcity, ethical constraints, donor variability, and difficulties in long-term culture. Furthermore, conventional cultures lack the compartmentalization needed to study sensory neuron processes and fail to recapitulate the synaptic connectivity between sensory and spinal cord neurons, limiting their translational relevance.

To address these limitations, we utilized a microfluidic platform enabling compartmentalized culture of human induced pluripotent stem cell (hiPSC) derived sensory neurons (hiPSC-SNs) to study the function of their processes. We also demonstrate the feasibility of microfluidic co-cultures of hiPSC-SN with human iPSC-derived cortical excitatory neurons (hiPSC-CNs) as a basis for future development of models for sensory-to-CNs communication circuit.

Using optimized protocols, we maintained stable microfluidic cultures and confirmed expression of pain-relevant sodium channels (Nav1.7, Nav1.8) in hiPSC-SN in both mono- and co-culture configurations. Leveraging this compartmentalized platform, we demonstrate that pharmacological blockade of Nav1.7 and Nav1.8 inhibits signal propagation along sensory neuron processes. We also demonstrate that growth factors modulate excitability of these processes.

This functional validation underscores the platform’s capability to investigate signal transmission along human sensory processes and demonstrates its potential for modelling more complex cellular interactions. Thus, we present a human iPSC-based microfluidic culture model that enables detailed study of sensory neuron processes and assessment of analgesics targeting nociceptive transmission, offering a significant advance toward analgesic drug discovery.

## Graphical abstract

**Graphical Abstract:**
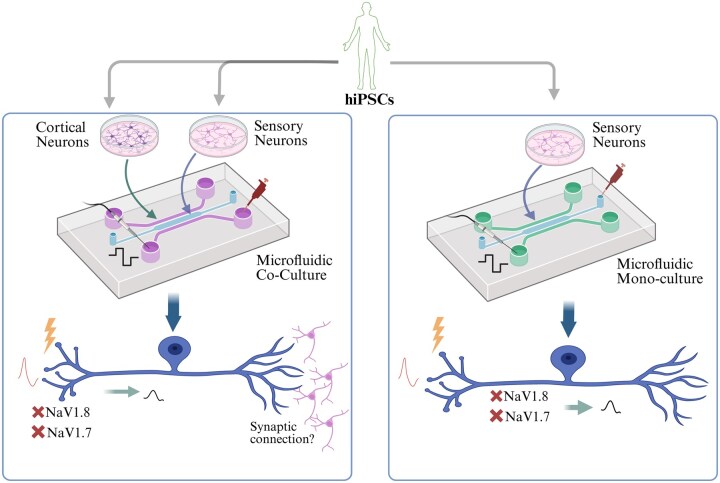

Significance statement The drug discovery process of analgesics is often constrained by a lack of human in vitro models that can specifically target the nerve fibers responsible for pain signal transmission. To overcome this limitation, we have established a model using human stem cell-derived sensory neurons in a microfluidic culture platform that allows compartmentalization of the cell soma and the signal transmitting processes. We use this system to demonstrate that signal transmission can be selectively blocked by targeting channels that contribute to signaling in these fibers. This model provides a transformative tool for screening potential analgesics and studying human pain mechanisms directly.

## Introduction

Latest estimates suggest that 20%-30% of the global population suffers from some form of chronic pain including high impact pain^1^ but the current pain treatments are often ineffective or associated with severe side effects.^2^ Evaluation of analgesic efficacy in drug discovery has relied primarily on rodent-based pre-clinical models, but they have shown poor translational success, leading to high failure rates in clinical trials.^3^^,^^4^ This translational gap may be due to species differences in nociceptor populations and pain-related ion channel expression, such as voltage-gated sodium channels.^5^ Therefore, human-relevant in vitro models offer a promising alternative to improve understanding of human molecular pain mechanisms and advance analgesic drug discovery.^6^

Primary human dorsal root ganglia (DRG) neurons, which transmit pain signals to the spinal cord neurons, are considered to have higher physiological relevance, but are difficult to obtain due to their limited availability. Human induced pluripotent stem cells (hiPSC) offer an alternative, as they can be differentiated into functional sensory neurons (hiPSC-SNs), expressing key nociceptor pain targets such as Nav1.7, Nav1.8, TRPA1 and TRPV1.^7^^,^^8^ However, conventional hiPSC-SN cultures have significant limitations in that they fail to capture the physiological complexity of pain signaling. They lack the compartmentalization needed to separate cell bodies from their processes, which is fundamental to understanding how nociceptive signals are carried from the periphery to the central nervous system.^9^ We have shown that microfluidic culture systems overcome these limitations by providing a structured environment that allows processes to be physically separated from cell bodies, enabling targeted pharmacological studies solely on sensory axonal function.^10^^,^^11^ Small, well-defined chambers enable independent manipulation of axonal and cell body environments across distinct compartments. This setup allows for localized drug application, enables the investigation of axon-specific signaling, supports synaptic connectivity, and better recapitulates the anatomical organization of peripheral pain system.

Successful generation and propagation of action potentials in nociceptive processes rely on voltage-gated sodium channels. Nav1.7 and Nav1.8 are critical mediators of transmission of pain signals by DRG neurons.^12–15^ These channels are implicated in inherited neuropathic pain syndromes, making them prime targets for analgesic drug discovery.^14^^,^^16–18^ We used selective sodium channel inhibitors VX-150 and SSCI-2 to investigate functional expression of Nav1.8 and Nav1.7, respectively, in the processes of hiPSC-SNs in microfluidic cultures. The potency and specificity of SSCI-2 and VX-150 as sodium channel inhibitors have been well-documented,^19^^,^^20^ with VX-150 showing promising results in human clinical trials.^21^ More recently selective Nav1.8 inhibitor VX-548 (Suzetrigine) has been approved by FDA for acute pain treatment.^22^ Although SSCI-2 has demonstrated robust efficacy in a noxious heat-evoked non-human primate model,^19^ there are currently no selective Nav1.7 inhibitors in clinical development, and the most advanced selective inhibitor, PF-05089771 developed by Pfizer, failed to reach statistically significant analgesic effects in a Phase 2 human clinical trial.^23^

As well as interacting with their targets in the periphery, sensory neurons form excitatory synapses on spinal dorsal horn neurons where pain signals are processed and transmitted to the brain.^24^ Hence, we sought to demonstrate the feasibility of building more complex culture models based on iPSC-derived neurons and to recapitulate these interactions in a microfluidic model. However, as primary human spinal cord neurons are extremely difficult to obtain and the protocols for differentiating human iPSC-derived spinal cord neurons are still under development, we chose the well characterized human iPSC-CNs instead. We hypothesized that hiPSC-CNs might provide a surrogate for excitatory DH neurons. While direct synaptic connections between these neurons are not known, studies have shown that co-culturing rodent peripheral sensory and cortical neurons leads to increased axonal and connective outgrowth in cortical neurons.^25^

Here, we demonstrate the viable differentiation and maintenance of hiPSC-SNs in monoculture and in co-culture with hiPSC-CN using microfluidic platform. We demonstrate that in both co- and monocultures of iPSC-SNs, inhibiting Nav1.8 with VX-150 or Nav1.7 with SSCI-2 significantly reduces action potential transmission in processes. Interestingly, the magnitude of this effect was dependent on the location of the application of the inhibitors along the length of the processes. Although the existence of synaptic connections between the hiPSC-CNs and hiPSC-SNs could not be conclusively confirmed, our results demonstrate that microfluidic cultures of hiPSC-SNs provide a functional model for studying the physiology of human sensory processes, which could be used for screening compounds for antinociceptive efficacy.

## Methods

### Reagents

Cal520 (AAT Bioquest) stock was dissolved in Pluronic acid and DMSO at 4 µM. SSCI-2 and VX-150 (both provided by Grünenthal) were dissolved in DMSO. All working solutions contained 0.1% DMSO. All stock solutions were kept at −20 °C and diluted to the working concentration on the day of the recording. For the synaptic experiments, the following compounds from Tocris were utilized to inhibit the synaptic transmission: D-AP5 (APV), CNQX disodium salt and SR 95531 hydrobromide (Gabazine). Each reagent was dissolved in distilled water at the following concentrations: 50 mM (D-AP5), 5 mM (CNQX disodium salt), and 15 mM (Gabazine) and were kept at –20 °C until use.

### Preparation of microfluidic devices

**Figure 1.**
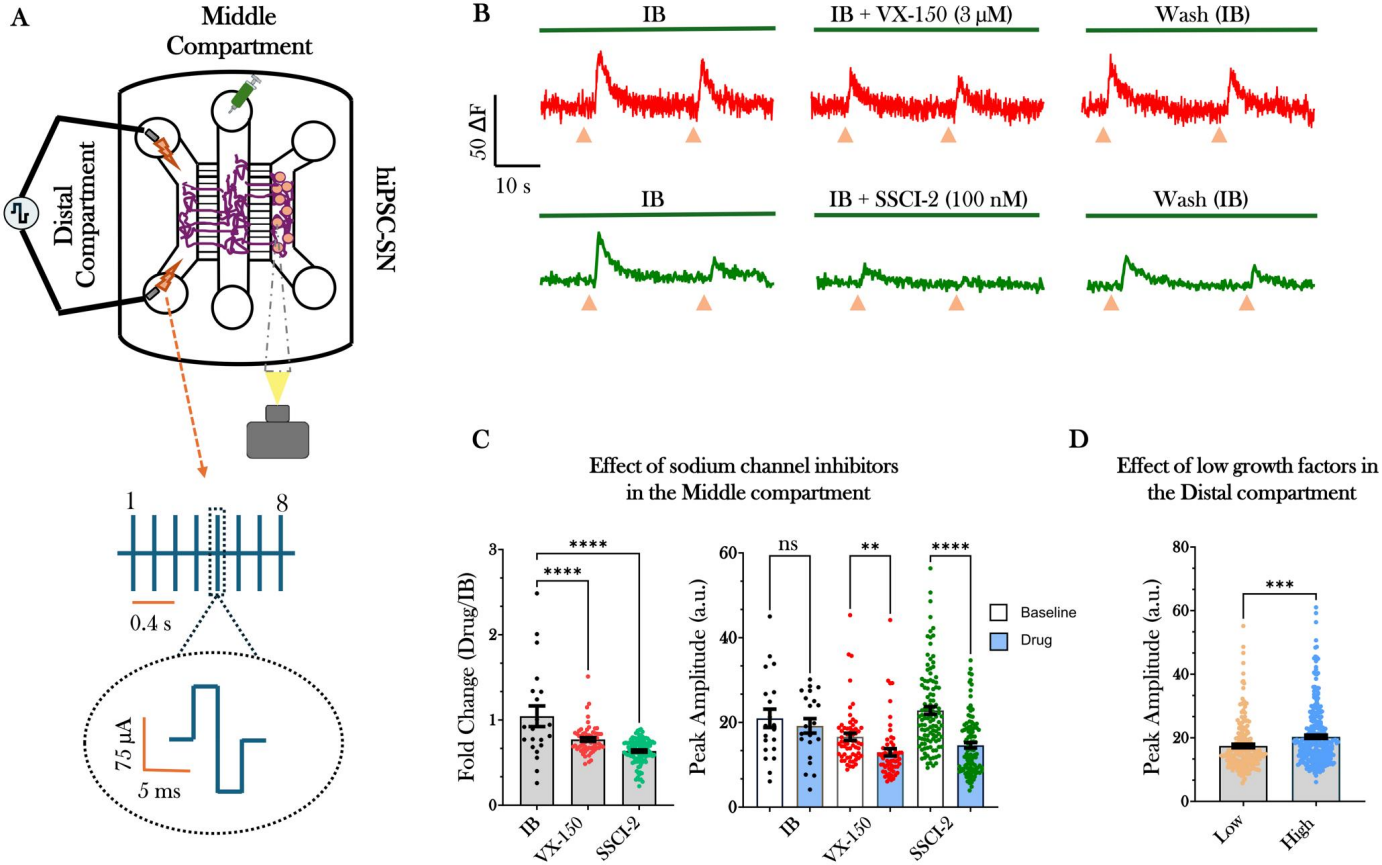
Functional properties of human-induced pluripotent stem cell (hiPSC)-sensory neuron (SN) processes in monocultures. (A) Schematic of the experimental setup (top). SSCI-2 or VX-150 was applied to the middle compartment , while the distal compartment was electrically stimulated with a train of 8 biphasic pulses at 5 Hz and 75 µA for 1 s (bottom). Ca^2+^ imaging was performed on hiPSC-SN soma. (B) Representative traces of Cal520 fluorescence intensity recorded from hiPSC-SN soma before and during application of VX-150 or SSCI-2. The distal compartment was stimulated twice (marked with arrows) at 2-min intervals before each buffer exchange (IB—imaging buffer). (C) Treatment of the middle compartment with either SSCI-2 or VX-150 led to a significant reduction of the cell response to electrical stimulation of the distal compartment. Comparison of the fold change (left) and peak amplitudes (right) in responses from vehicle, SSCI-2 (100 nM) or VX-150 (3 µM) treated plates One-way analysis of variance, *P-*value: <.0001; *N* = 21, 61, and 114 for Vehicle, VX-150 and SSCI-2, respectively. Data collected across 5 devices from 2 independent differentiation rounds. Experiments were performed at DIV28. (D) The effect of growth factor concentrations on soma responses to stimulation of the distal processes. From DIV14 until DIV28, the distal compartment was treated with either high (4-fold higher concentrations than the soma) or low (same concentrations as the soma compartment). Soma responses to electrical stimulation of distal processes showed an increase with high growth factor treatment (Welch’s *t*-test, *P* = .0002; *n* = 203, 318 respectively. Data collected across 4 devices from 2 independent differentiation rounds). *, *P* < 0.05; **, *P* < 0.01; ***, *P* < 0.001; ****, *P* < 0.0001.

The protocol used is a modification of the protocol described by Tsantoulas et al.^10^ Three-compartment microfluidic silicone devices (TCND1000; Xona Microfluidics, Figure 1A) were used. Microgrooves measuring 10 µm in width, 3 µm in height, and 500 µm in length separated the 2 lateral compartments from the middle compartment in the microfluidic devices. All 3 compartments were 100 µm in height. The middle compartment was 1000 µm in width. The devices were prepared according to previous published protocols.^11^ Briefly, Glass bottom dishes (WillCo Wells) were coated with 0.1 mg/mL poly-l-lysine (Sigma) and dried overnight. Microfluidic devices were washed, dried overnight and assembled onto the glass as described previously.^10^ The assembled devices were then treated with 40 µg/mL laminin (Sigma) dissolved in PBS (Thermo Fisher) for 4 hours.

### Cell culture

Human iPSC-derived sensory neuron progenitors (ax0055) and human iPSC-derived neural stem cells (ax0015) were purchased from Axol Bioscience Ltd. No additional ethical approval was required for this study. Cell culture media were obtained from Axol Bioscience Ltd. All batches of the iPSCs originated from the same donor (male, cord blood). The corresponding plating, differentiation and maintenance media were prepared according to the manufacturer’s instructions, unless otherwise stated below. For co-cultures cells were seeded simultaneously in separate compartments of assembled microfluidic devices: iPSC-derived sensory neuron progenitors were seeded at 90 000 cells in the middle compartment and iPSC-derived neural stem cells at 50 000 cells in the side compartment of a microfluidic device. For the monocultures of hiPSC-SNs, 90 000 sensory neuron progenitors were seeded in one side compartment of the microfluidic devices. During differentiation and maintenance of the co-cultures, fluidic isolation was preserved by maintaining a hydrostatic pressure differential between compartments, using 200 µL in the sensory compartment and 150 µL in the cortical compartment (maximum well volume 200 µL). The cultures in microfluidic devices were maintained at 37 °C and 5% CO_2_ in humidified incubators under normoxic atmospheric O_2_ conditions. Manufacturer’s protocols for differentiation and maintenance of iPSCs were followed with the following modifications: iPSC-derived sensory progenitors were treated with 0.5  µg/mL Mitomycin C at DIV4 for 1.5 hours.

In order to promote neurite outgrowth, support consistent crossing of the processes into the distal compartments, and improve cell health, the middle and distal compartments in monocultures and the distal compartment in co-cultures were treated with increased concentration of growth factors (Table S1) from DIV 14 onward. This treatment continued until the end of the experiments. Experiments were carried out between DIV 21 and DIV28 (days in vitro), as recommended by the manufacturer.

### Calcium imaging

At DIV21-28, all 3 microfluidic compartments of the microfluidic devices were incubated with 4 μm calcium indicator dye Cal520 (AAT Bioquest) for 2 hours at 37 °C in imaging buffer consisting of Ca^2+^ and Mg^2+^-free HBSS (Sigma) supplemented with 10 mM HEPES (Thermo Fisher), 2 mM CaCl_2_ (Sigma), 1 mM MgCl_2_, and 2 mM probenecid (Sigma). The pH was adjusted to 7.4 with NaOH. The devices were mounted on an inverted fluorescent microscope (Nikon Eclipse TE200) equipped with 10X or 20X Plan Fluor 0.5 NA objective (10X magnification was used for sensory recordings, and 20X magnification was used for recording from cortical neurons) connected to EasyRatioPro calcium imaging system (Horiba Scientific). Fluorescent image sequences were acquired using an ORCA 4.2 sCMOS camera (Hamamatsu) with 492 nm excitation and 510 nm emission at 6 Hz.

In each recording phase, 2 stimulation trains were delivered 2 minutes apart. For each stimulation, a 30 seconds pre-stimulation period was recorded and used as the baseline for response analysis. Electrical stimulation was delivered to the distal compartment using a NeuroLog modular system (Digitimer). The system was configured to generate trains of biphasic pulses using 2 parallel NL800A Current Stimulus Isolators (Digitimer) to deliver the cathodic and anodic phases of each pulse through electrodes positioned in the distal compartment (Figure 1A). Each stimulus train consisted of 8 biphasic pulses delivered at 5 Hz, with an amplitude of 75 µA and a duration of 5 ms per pulse (2.5 ms per phase). Ca^2+^ responses were recorded from the cell soma in the sensory or cortical compartment, as appropriate.

Drug application was performed by complete buffer exchange, replacing the buffer in the relevant microfluidic compartment with buffer containing the specific blocker. Attention was paid to preserve fluidic isolation during recordings by maintaining a 150 µL liquid volume differential between treated and adjacent untreated compartments. Control responses (vehicle) to electrical stimulation of the distal compartment were recorded, followed by pre-application of the blockers for 3 minutes. The drugs were then washed out with imaging buffer and responses to stimulation of the distal compartment were recorded again. In all experiments, somal responses to application of 40 mM KCl to the soma compartment were recorded at the end of the recordings. All experiments were carried out at room temperature (RT) and all solutions were equilibrated to RT before application during the recording. Data were collected from minimum of 2 independent differentiations batches (biological replicates) from multiple plates (technical replicates) across the batches.

### Image analysis

Image sequences were imported into FIJI (ImageJ) for analysis of cellular responses. After image stabilization using the FIJI Image Stabilize plugin, soma responses to 40 mM KCl applied at the end of each recording were used to define regions of interest (ROIs). Responding soma were identified using the FIJI Threshold tool with a manual threshold of 5%-7%, after which the Particle Analysis module was used to generate ROIs using a particle size range of 5-50 pixels. The average fluorescence intensity of each ROI over time was extracted.

### Immunofluorescence labeling

To reduce cell clumping, the cortical neuron progenitors were seeded and differentiated separately in 24 well plates with PDL/Laminin coating (0.1 mg/mL poly-l-lysine and 40 µg/mL laminin). In parallel, hiPSC-SN progenitors were maintained in microfluidic devices. At DIV14, the cortical cells were dissociated with Accutase for 10 minutes and seeded into the side compartment of the microfluidic device, adjacent to sensory neuron compartment.

Media was removed from all wells in microfluidic devices and washed once with calcium and magnesium containing HBSS. Cells were fixed with 4% PFA in PBS (MERCK) at RT for 10 minutes. Next, cells were washed 3 times with HBSS for 5 minutes. HBSS was removed and cells were incubated in HBSS with 0.2% PBS-Tritan X, and the primary antibodies: chicken Peripherin (1:1000, # PA1-10012 Thermo Fisher) and rabbit MAP2 (1:500, #Ab32454 Abcam) primary antibodies. Cells were incubated with the primary antibody for 1 hours at RT. The cultures were then incubated with secondary antibody solution containing HBSS and Alexa 488 anti-chicken (#A11039), Alexa 633 anti-rabbit (A21070) antibodies and DAPI (1:1000) for 1 hours at RT.

### RNAscope *in situ* hybridization and immunofluorescence

The cells were washed with 1xPBS, fixed consecutively in 2% and 4% PFA for 5 minutes each. After washing with 1xPBS, the cells were dehydrated in ascending ethanol series and stored at −20 °C until labeling. The microfluidic chambers were detached and RNAscope (multiplex fluorescent v2 kit) was performed as instructed by Advanced Cell Diagnostics (ACD-bio). The labeling was performed following the “RNAscope Multiplex Fluorescent v2 Assay for Cultured Adherent Cells” protocol. RNAscope probes against SCN9A (Catalog No.: 562251-C1), SCN10A (Catalog No.: 406291-C2) and fluorescent TSA dyes (520 and 570) were used. For immunocytochemistry, the cells were permeabilized with 0.1% (v/v) Triton X-100 (#X100, Merck) and blocked with 5% (w/v) bovine serum albumin (Merck) for 1 hour. To label neurons, primary antibody for ß-3 Tubulin (1 µg/mL, R&D systems) incubated overnight in blocking solution at 4 °C. The cells were washed and secondary goat anti-mouse Alexa Fluor 488 (4 µg/mL, Invitrogen) was incubated for 1 hour. After washing, the glass coverslips are mounted on petri dishes with Aqueous Fluoroshield with DAPI (Abcam).

Imaging was performed with confocal Zeiss LSM900 at ×20 magnification (Plan-Apochromat ×20/0,8 M27). Image acquisition and processing were performed on ZEN software (v3.10).

### RNA isolation and real-time quantitative PCR

Total RNA was isolated using RNeasy Micro Kit (Qiagen) from cortical and sensory compartments of microfluidic devices at DIV21 and DIV27 of maturation according to manufacturer’s protocol. Total RNA concentration and purity were analyzed by the automated electrophoresis tool, TapeStation 4200 for quantity and quality control of RNA and DNA (Agilent). For this the High Sensitivity RNA Screen Tape kit was used (Agilent, Screen Tape Cat. 5067-5579, Screen Tape Sample Buffer Cat.5067-5580, Screen Tape Ladder Cat.5067-5581). RNA obtained was reverse transcribed to single-stranded cDNA in 20 μL of reaction mixture with RNase inhibitor using the SuperScript IV VILO Master Mix with ezDNase (Invitrogen by Thermo Fisher Scientific, Cat.11766500). For transcription the Mastercycler X50s (Eppendorf) was used. Expression of target genes and endogenous control (GAPDH and GUSB) mRNA was assessed by real-time quantitative PCR using the TaqMan gene expression assays on a QuantStudio 6 Flex real-time PCR system (Applied Biosystems, Darmstadt, Germany) using the TaqMan fast advanced master mix (Applied Biosystems, Cat.4444557) according to the manufacturer’s recommendations. The Ct method was used to determine expression levels for each gene of interest by normalizing the quantified mRNA to GAPDH and GUSB. Primers were obtained from Thermo Fisher TaqMan Gene Expression Assay catalog. The utilized primers are GAPDH (Hs99999905_m1), GUSB (Hs00939627_m1), NaV 1.7 (Hs01076711_m1), and NaV 1.8 (Hs01045137_mH).

### Data analysis

The resulting Cal520 fluorescence-intensity traces were visualized and analyzed using Clampfit software (Molecular Devices). Traces were excluded from analysis if the cell failed to respond to either stimulation under imaging buffer (control), failed to show at least one response during the wash recording set, showed recording artifacts, or if cell movement resulted in artifacts or loss of ROI. Traces were also excluded if baseline shifts after drug or imaging buffer application exceeded 10%. All responses were assigned by visual inspection with an amplitude of at least 4 standard deviations above the signal average. No formal blinding was used during treatment allocation or data analysis. ROI selection, trace exclusion, and response analysis were performed according to predefined criteria.

The amplitude of each response was determined by calculating a 30 seconds baseline prior to cell stimulation and subtracting from maximal response achieved within 2 minutes of stimulation. The peak amplitudes of the responses to successive stimulations during each drug application were averaged and used to compare to the responses in control (imaging buffer) conditions. Fold change in response amplitude for each individual cell was used as the primary quantitative measure. To account for the substantial heterogeneity in responses to the blockers, a predefined 40% inhibition threshold was used to quantify the proportion of more strongly inhibited cells. Fold change in response was calculated as the average of the drug-treated response amplitudes divided by the average of the baseline response amplitudes. A reduction of the fold change greater than 40% was used as a threshold for drug-induced inhibition. All statistical analysis was carried out using GraphPad Prism 10 (GraphPad Software LLC). All data are presented as mean ± SEM unless otherwise stated. Welch’s *t*-test was used for comparison of the response amplitudes between 2 independent groups, One-way analysis of variance (ANOVA) was used for comparisons of fold change responses between treatment groups, 2-way repeated measure ANOVA was used for the analysis of changes in response amplitude, Fisher’s exact test was used to compare the proportion of cells exceeding the predefined operational 40% inhibition threshold, and Kolmogorov–Smirnov tests were used to compare distributions of fold change responses. Statistical tests were selected a priori according to the type and distribution of the data. Statistical significance was set at *P* < .05. The effect sizes are reported as estimated mean difference in fold change between conditions, with their respective 95% confidence intervals (CIs).

## Results

### Block of Nav1.7 and Nav1.8 channels in hiPSC-SN processes inhibits signal transmission in microfluidic monocultures

Generation and propagation of action potentials rely on voltage-gated sodium channels. Nav1.7 and Nav1.8 sodium channels are critical mediators of transmission of pain signals in nociceptive processes,^12–14^ making them prime candidates for therapeutic intervention in nociceptive models. To investigate the role of Nav1.8 and Nav1.7 in initiation and propagation of action potential in human sensory neurons, we optimized protocols for a microfluidic culture of hiPSC-SNs. Human iPSC-derived sensory neuron progenitor cells (see Methods) were seeded in the microfluidic devices (Figure 1A) and were maintained for up to 29 days to differentiate into sensory neurons. We observed robust growth and arborization of the processes in microfluidic cultures with neurites crossing along the microfluidic channels into the adjoining compartments (Figure S1, see online supplementary material for a color version of this figure).

We then investigated responsiveness of hiPSC-SN processes using electrical stimulation in the distal compartment (Figure 1A). We had previously demonstrated that electrical stimulation of processes in the distal compartments of microfluidic cultures of DRG neurons generates action potentials that propagated along the processes to the soma, leading to an increases in the somal Ca^2+^ concentration and can be used as a readout of axonal function.^10^^,^^11^ Here we found that stimulation of hiPSC-SN processes led to a robust increase in the somal Ca^2+^ concentration (Figure 1A and B).

We used SSCI-2 and VX-150, selective inhibitors of Nav1.7 and Nav1.8, respectively, to investigate their effect on the propagation of action potentials along sensory neuron processes in microfluidic cultures. We found that applying SSCI-2 (100 nM) to the middle compartment (Figure 1A) reduced amplitude of soma responses to stimulation of distal processes by 36% ± 1% (mean difference in fold change vs. IB: 0.27 [95% CI: 0.15-0.40]) (Figure 1B and C). Addition of VX-150 (3 µM) to the middle compartment led to a reduction by 26% ± 2% (mean difference in fold change vs. IB: 0.41 [95% CI: 0.30-0.53]) in the soma responses to electrical stimulation of the distal processes (Figure 1C). The comparison of the peak intensity amplitudes demonstrated no change with application of buffer (estimated mean difference vs. baseline [arbitrary units.]: −1.79 [95% CI: −6.06 to 2.49]) but a significant reduction in amplitude in response after treatment with VX-150 (mean difference vs. baseline [arbitrary units] − 3.66 [95% CI: −6.17 to −1.12]) or SSCI-2 (mean difference vs. baseline [arbitrary units]—8.23 [95% CI: −10.09 to −6.38]). These results demonstrate that application of either Nav1.7 or Nav1.8 inhibitors is sufficient to impede the propagation of signals in hiPSC-SN processes in microfluidic monocultures, consistent with the functional expression of Nav1.7 and Nav1.8 sodium channels in hiPSC-SNs processes.

### Effect of the concentration of growth factors on the responses of hiPSC-SN distal processes in microfluidic monocultures

The differentiation and growth of sensory neurons, both in vivo and in hiPSC-SN in vitro cultures, depend on a combination of growth factors including NGF, BDNF, NT3, and GDNF.^7^^,^^8^ We had shown previously that concentration of growth factors was a key determinant of the axonal health and arborization to promote traversing into the adjoining chambers in microfluidic cultures.^10^^,^^11^^,^^26^ Consequently, we used a higher concentration of growth factors in the distal compartment than the somal compartment of the microfluidic monocultures (see methods, Table S1). However, we sought to investigate whether lowering the concentration of the growth factors in the distal compartment to the same basal level as the somal compartment (Figure 1A) would negatively impact the responses to the stimulation of the distal processes^27–33^ We observed that 14 days of treatment (DIV14-DIV28) with lower concentrations of NGF, BDNF, GDNF, and NT-3 in the distal compartment led to a modest but significant decrease in the peak amplitude response of hiPSC-SN soma to electrical stimulation of the distal processes (mean difference [a.u.]: 2.88 [95% CI: 1.407-4.355]) (Figure 1D). These data demonstrate that increased concentration of growth factors in the distal process compartment relative to the soma compartment promotes greater responsiveness of sensory processes in the microfluidic cultures of hiPSC-SNs. We therefore also adopted this treatment protocol for all subsequent experiments.

### Microfluidic cocultures of human iPSC–derived sensory and cortical neurons

We next established microfluidic co-cultures of hiPSC-SNs and hiPSC-CNs to assess sensory neuron process function in a more complex human cellular environment.

**Figure 2.**
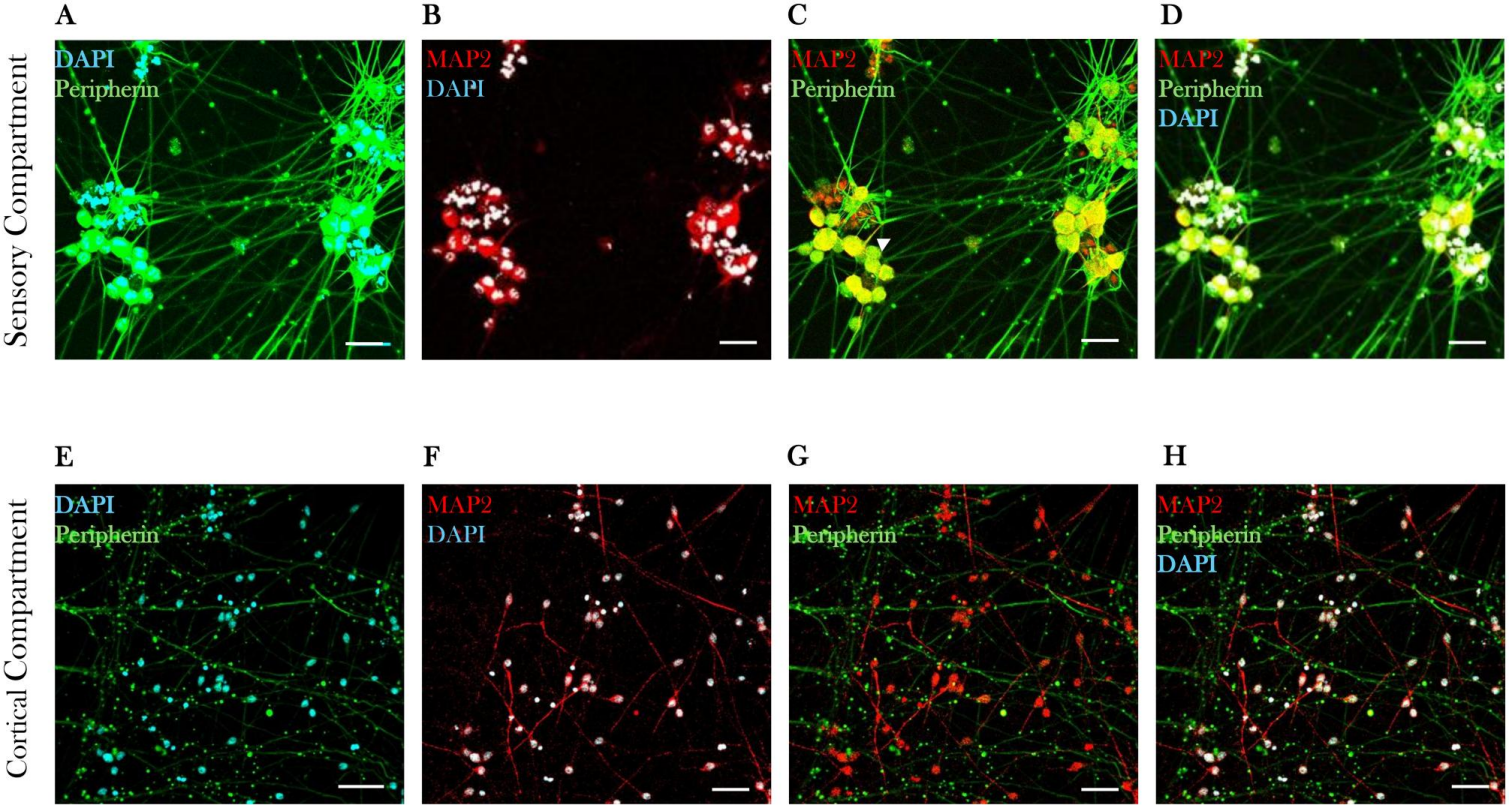
Expression of peripherin and MAP2 in cocultures. Cocultures were fixed and stained at DIV27. Representative immunofluorescence images of the sensory and cortical neuron compartments are shown for peripherin (green), MAP2 (red), and DAPI (cyan). (A-D) Sensory compartment: overlap of peripherin and DAPI (A), MAP2 and DAPI (B), MAP2 and peripherin (C), and all 3 markers (D). hiPSC-SN soma show positive staining for both peripherin and MAP2. Arrowheads indicate costaining of proximal sensory neurites with peripherin and MAP2 observed occasionally. Peripherin-positive distal processes are negative for MAP2 in the hiPSC-SN compartment. (E-H) Cortical compartment: overlap of peripherin and DAPI (E) and MAP2 and DAPI (F). Peripherin-positive processes are negative for MAP2 (G, H). MAP2-positive hiPSC-CN soma and neurites are negative for peripherin (G, H). Scale bar, 40 µm.

Human neural stem cells were seeded in the adjacent chamber to the sensory neuron progenitor cells and allowed to differentiate into excitatory cortical neurons (hiPSC-CNs) alongside the sensory progenitor cells (see Methods). Both hiPSC-SNs and hiPSC-CNs were differentiated and maintained in microfluidic cultures according to the optimized protocols (see Methods). Extensive arborization and growth of neurites in culture were observed in both compartments (Figure 2). Robust Ca^2+^ responses to KCl-induced depolarization were observed in the cell bodies of both hiPSC-SN and hiPSC-CN neurons (Figure S2A and B, see online supplementary material for a color version of this figure).

We confirmed the expression of peripherin and MAP2 at DIV27 in the co-cultures (Figure 2). hiPSC-SNs express peripherin in the co-cultures (Figure 2A). MAP2 labeling was observed in the sensory soma and extended weakly into some proximal neurites close to the cell body (Figure 2B and C), whereas distal peripherin-positive neurites lacked detectable MAP2 signal (Figure 2A-D). On the other hand, the hiPSC-CNs soma and neurites were positive for MAP2 but negative for peripherin (Figure 2F-H). In addition to MAP2-positive cortical neurites, peripherin-positive/MAP2-negative neurites were observed in the cortical compartment, consistent with sensory neuron processes that had traversed the microgrooves from the adjacent hiPSC-SN compartment (Figure 2E-H).

As we observed hiPSC-SN processes traversing into the adjacent compartments in co-cultures, we next investigated whether these processes formed functional synapses with hiPSC-CNs. We have shown previously that in microfluidic co-cultures of rodent sensory and spinal cord neurons, stimulation of the distal sensory axons leads to the activation of spinal cord neurons, consistent with synaptic input from the same crossing sensory neurons.^11^^,^^26^ We assessed synaptic connectivity in co-cultures of hiPSC-SN and hiPSC-CNs, by stimulating the sensory processes in the distal compartment while recording responses in the iPSC-CNs compartment at DIV28 − 32 (Figure S3A, see online supplementary material for a color version of this figure). To verify that any responses in hiPSC-CNs were from synaptic contact with presynaptic hiPSC-SN processes, we applied mixture of CNQX, APV, and Gabazine to block synaptic transmission^11^ CNQX and APV block AMPA and NMDA receptors subtypes, respectively, abolishing excitatory transmission. We also included Gabazine to block GABA-A receptors and eliminate any potential GABAergic excitatory influence in the cultures.^34^ We only found a singular trace where the hiPSC-CN response to stimulation of hiPSC-SN processes was blocked to this mixture of the blockers (Figure S3B, see online supplementary material for a color version of this figure). No additional examples were observed at DIV28 or later time points, leading us to conclude that, under these culture conditions, hiPSC-SN processes do not efficiently form synaptic contacts with hiPSC-CNs.

### Human iPSC–derived sensory neurons express Nav1.7 and Nav1.8 mRNA in a compartmentalized coculture model system with human iPSC–derived cortical neurons

**Figure 3.**
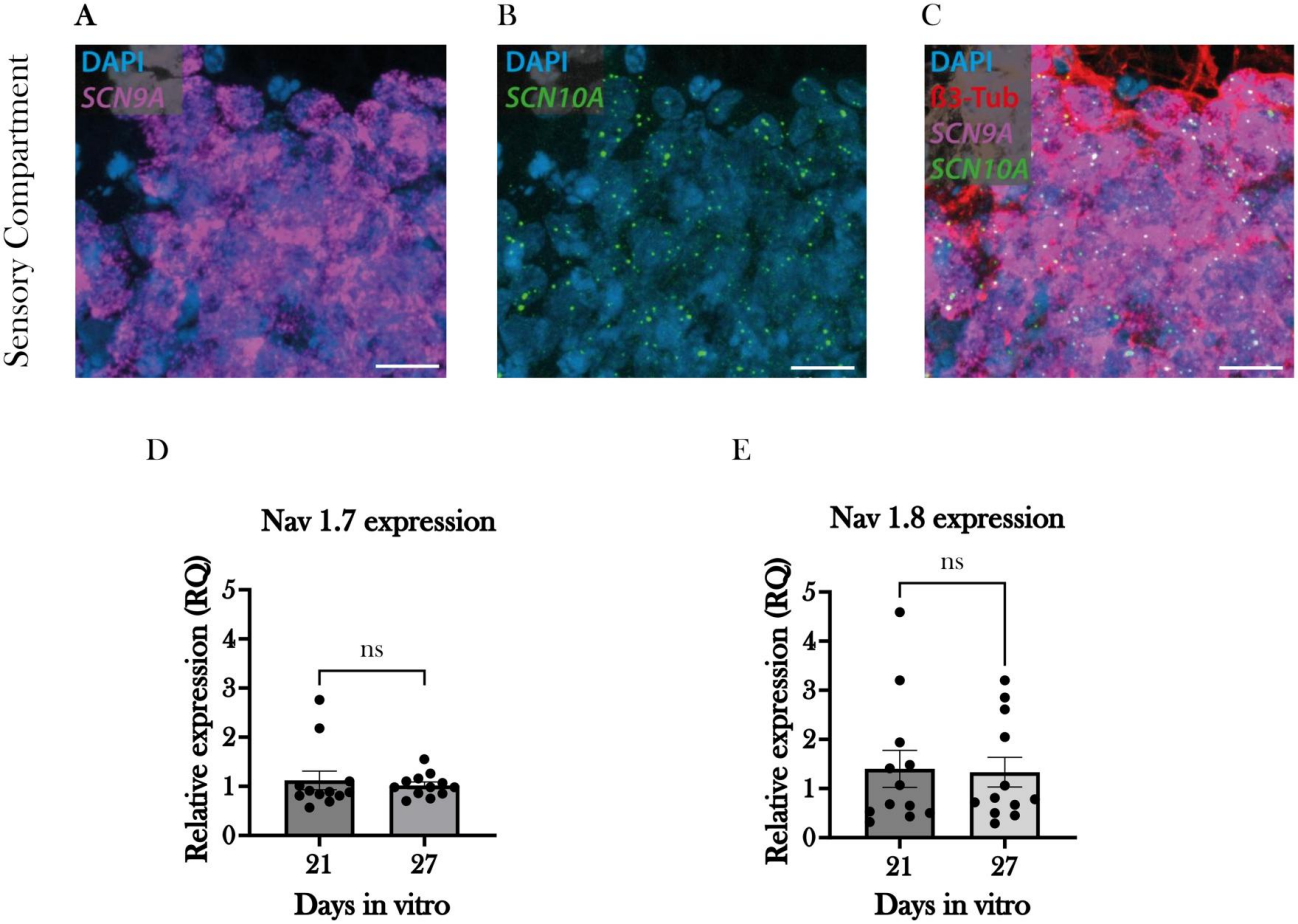
Expression of *SCN9A* and *SCN10A* mRNA within human-induced pluripotent stem cell (hiPSC)-SN soma in coculture hiPSC-CN. (A-C) mRNA labeling of hiPSC-SN via RNAscope at DIV27 with *SCN9A* (magenta) and DAPI (cyan) (A), *SCN10A* (green) and DAPI (B) and overlapped with β3-tubulin expression (C). Clear expression of *SCN9A* is observed in the sensory neurons, with sparser *SCN10A* expression throughout the neurons. (D, E) Relative mRNA expression for both *SCN9A* and *SCN10A* was measured in the iPSC-derived sensory neurons at 2 different time points in coculture using qPCR. No difference in the expression levels for either mRNA was found between DIV21 and DIV27 (*P-*value .6211and .8897, respectively). For each time point, samples were prepared from the hiPSC-SN soma compartments of 4 independent microfluidic cultures plates and analyzed in technical triplicates. Scale bar 50 µm.

We then sought to determine whether hiPSC-SNs in co-culture express Nav1.7 (*SCN9A*) and Nav1.8 (*SCN10A*) mRNA. To evaluate the cellular abundance of *SCN9A* and *SCN10A*, we utilized RNAScope to measure mRNA expression in sensory and cortical neurons at DIV27 of maturation (Figure 3A-C and Figure S5, see online supplementary material for a color version of this figure). We observed robust expression of *SCN9A* in the hiPSC-SN compartment (Figure 3A). *SCN10A* was also detectable, albeit at lower levels, in these neurons (Figure 3C). In the cortical compartment, low to sparse SCN9A and SCN10A signals were observed under the imaging conditions used (Figure S5, see online supplementary material for a color version of this figure).

Previous reports suggest that the expression of *SCN9A* and *SCN10A* mRNA is low early during differentiation but increases as the culture matures.^7^ We used qPCR to quantify and assess the levels of *SCN9A* and *SCN10A* mRNA at DIV21 and DIV27 (Figure 3D and E). We found that hiPSC-SNs express *SCN9A* and *SCN10A* at both timepoints, but the expression levels did not significantly change with additional time in culture (change in relative expression: −0.10 [95% CI: −0.52 to 0.32] and −0.07 [95% CI: −1.07 to 0.93], respectively). These data demonstrate transcription of the 2 key nociceptive markers *SCN9A* and *SCN10A* in hiPSC-SNs in co-culture with hiPSC-CNs after DIV21.

### Functional expression of Nav1.7 and Nav1.8 channels in hiPSC-SN processes cocultured with hiPSC-CNs

We further investigated the impact of Nav1.8 and Nav1.7 inhibitors on signaling in co-cultures of hiPSC-SN and hiPSC-CN, providing evidence of functional contribution of Nav1.7 and Nav1.8 channels to signal propagation at DIV21-22 and DIV25-28.

We first tested the Nav1.8 inhibitor, VX-150 (1 and 3 µM), and the Nav1.7 inhibitor, SSCI-2 (50 and 100 nM), for their ability to inhibit signal transmission in microfluidic co-cultures. For these experiments, we applied the inhibitors in both the distal and hiPSC-SN soma compartments (i.e., the middle compartment, Figure S4A, see online supplementary material for a color version of this figure). There was a slight but significant effect of the higher concentration of VX-150 on the average amplitude of the response (Figure S4B, see online supplementary material for a color version of this figure). At higher concentration of VX-150, but not SSCI-2, we also observed an increase in the number of cells showing >40% reduction in response amplitude (Figure S4C and D, see online supplementary material for a color version of this figure). We therefore selected the higher concentration of the inhibitors for the remaining experiments.

**Figure 4.**
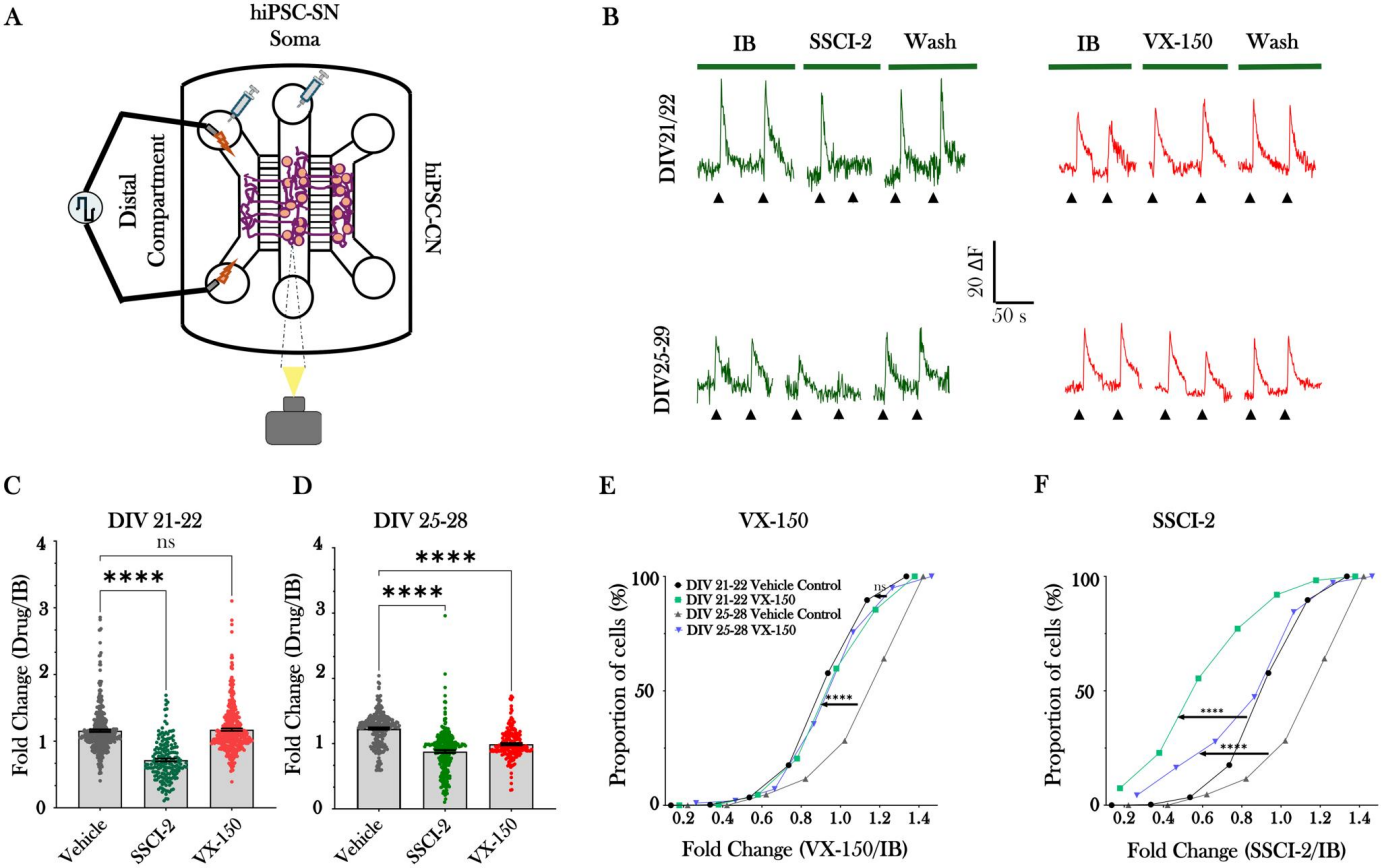
Effect of selective sodium channel inhibitors VX-150 and SSCI-2 on hiPSC-SN processes during maturation. (A) Schematic of the experimental setup. VX-150 or SSCI-2 was applied to both the distal and the sensory soma compartments. The distal compartment was electrically stimulated and stimulus-induced responses were recorded by Ca^2+^ imaging from the human-induced pluripotent stem cell (hiPSC)-SN soma compartment. (B) Representative traces of Cal520 fluorescence intensity changes in the hiPSC-SN soma in response to stimulation of the distal compartment under different conditions. Arrowheads indicate the application of the stimulation train to the distal compartment. (C) Comparison of the fold changes in hiPSC-SN responses after treatment with VX-150 or SSCI-2 at DIV21-22. Treatment with SSCI-2 led to a significant reduction in responses to electrical stimulation compared with vehicle control, whereas treatment with VX-150 did not. One-way analysis of variance (ANOVA), *P-*value: .7419 and <.0001; *N* = 394, 179, and 375 for vehicle, VX-150, and SSCI-2, respectively. (D) Treatment with either VX-150 or SSCI-2 at DIV25-28 led to a significant reduction in responses compared with vehicle control. One-way ANOVA, *P-*value: <.0001; *N* = 258, 264, and 204 for vehicle, VX-150, and SSCI-2, respectively. (E) Distribution of fold changes in responses in the presence of VX-150. No significant shift in the distribution was observed at DIV21-22, whereas at DIV25-28 the distribution shifted to the left compared with vehicle control, indicating an increased proportion of cells with reduced responses. Kolmogorov-Smirnov tests: *N* = 398, 397, 258, and 204; *P* = .2621 at DIV21-22 and *P* < .0001 at DIV25-28. (F) Distribution of fold changes in responses in the presence of SSCI-2. The distribution was shifted to the left compared with vehicle control at both time points. Kolmogorov-Smirnov tests: *N* = 398, 179, 258, and 204; *P* < .0001 for both time points. Data were collected from 12 plates at DIV21-22 and 12 plates at DIV25-28 across 5 independent differentiation batches. Data in (C) and (D) are presented as mean ± SEM. ****, *P* < 0.0001.

We found that at DIV21-22 of maturation, treating the soma and the distal processes of hiPSC-SNs with VX-150, 3 µM (Figure 4A and B) did not result in a significant reduction in the amplitude of the response to electrical stimulation of the processes in comparison to the vehicle control (mean fold change difference 0.02 [95% CI: −0.04 to 0.02]) (Figure 4C). On the other hand, treatment of the soma and processes with SSCI-2 at 100 nM (Figure 4B) resulted in significant reduction in the amplitude of the soma responses to stimulation of the distal processes at DIV21-22 (mean difference in fold change vs. vehicle: −0.44 [95% CI: −0.51 to −0.37) (Figure 4C). At DIV25-28, blocking Nav1.8 and Nav1.7 with VX-150 and SSCI-2, respectively, resulted in a significant reduction of the soma responses to stimulation of the distal processes (mean VX-150 fold change vs. vehicle: −0.24 [95% CI: −0.30 to −0.18], and mean SSCI-2 fold change vs. vehicle: −0.35 [95% CI: −0.41 to −0.30]) (Figure 4B and D). As Nav1.8 is only expressed by a subset of differentiated hiPSC-SNs (Figure 3B), we also examined the distribution of fold change in response to the presence of VX-150. We found no change in distribution of fold changes at DIV21 (Figure 4E), whereas at DIV25-28, there was a significant left-shift, indicating an increase in the proportion of cells with reduced responses due to the blocker effect (Figure 4F). Meanwhile, the fold change distribution for SSCI-2 displays a strong shift at both time points in comparison to the vehicle control (Figure 4E and F). While *SCN10A* mRNA levels remain stable over time in co-culture, these data are consistent with an increase in the functional contribution of Nav1.8 channels over time, particularly by DIV25-28. In contrast, both the mRNA levels and the functional contribution of Nav1.7 to signaling in the processes, appear to remain stable after DIV21.

### Application site–dependent differential effects of VX-150 and SSCI-2 on soma responses to stimulation of distal processes

**Figure 5.**
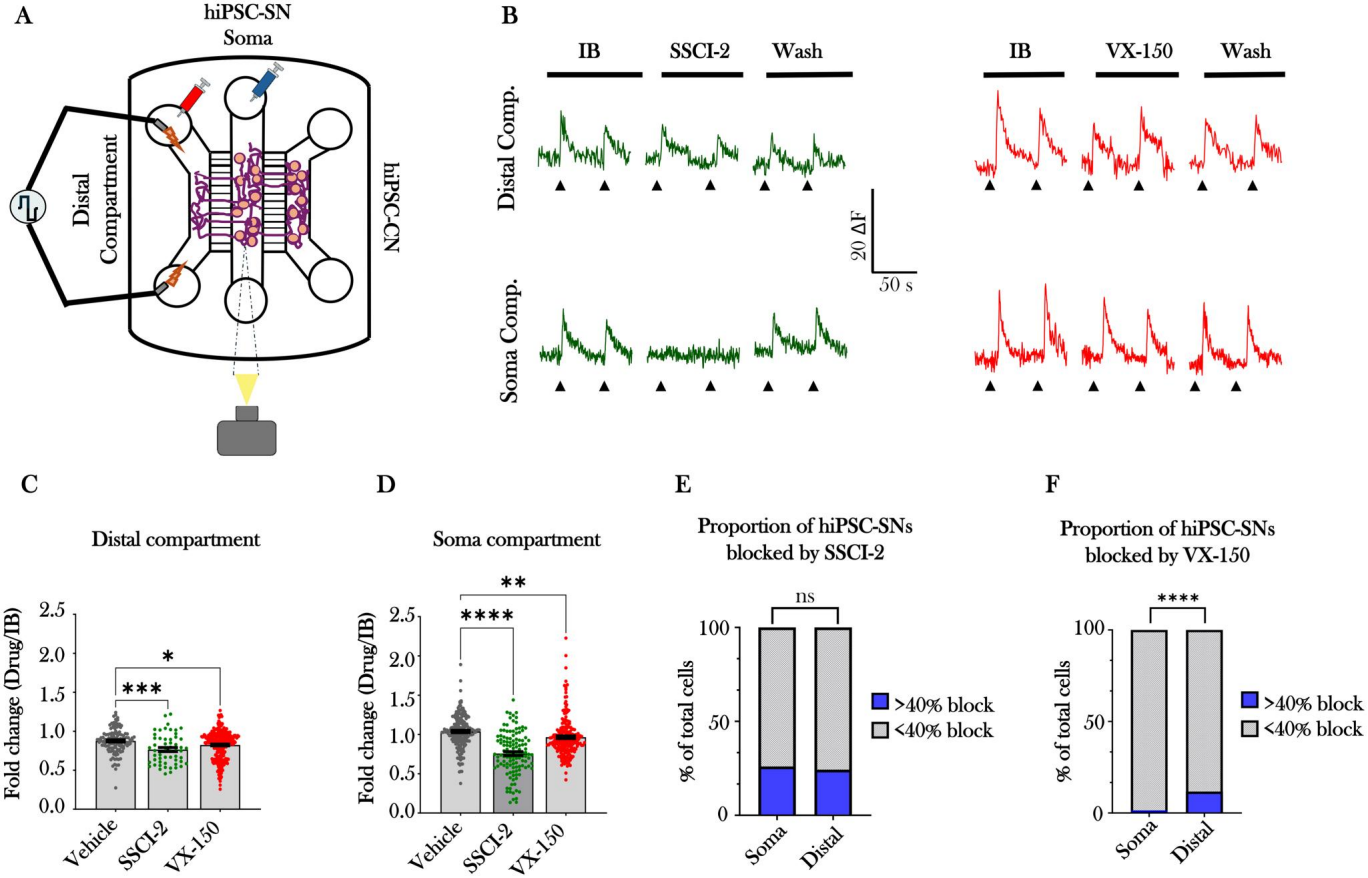
Location-dependent effects of SSCI-2 and VX-150 on responses to stimulation of human-induced pluripotent stem cell (hiPSC)-SN processes. (A) Schematic of the experimental setup. SSCI-2 or VX-150 was applied either to the distal or the sensory soma compartment. Ca^2+^ imaging was performed in the hiPSC-SN soma compartment while electrical stimulation was applied to the distal compartment. Experiments were performed at DIV28-29. (B) Representative traces of Cal520 fluorescence intensity changes in the hiPSC-SN soma in response to stimulation of the distal compartment under different conditions. Arrowheads indicate the application of the stimulation train to the distal compartment. (C, D) Comparison of changes in response after treatment with SSCI-2 or VX-150 applied either to the distal compartment (C) or the sensory soma compartment (D). Treatment with either SSCI-2 or VX-150 at either location led to a significant reduction in responses to electrical stimulation compared with vehicle control. One-way analysis of variances (ANOVAs), *P-*value: .002 and .0117 (C), <.0001 and .0044 (D); *N* = 133, 55, and 275 for C, and *N* = 213, 125, and 209 for D. (E) Location of SSCI-2 application did not significantly alter the proportion of cells with a reduction in response amplitude of 40% or more. Fisher’s exact test: *N* = 55, 125; *P* = .7138. (F) The proportion of cells showing a reduction in response amplitude of 40% or more following VX-150 application was higher when the distal compartment was treated. Fisher’s exact test: *N* = 346, 215; *P* < .0001. Data were collected from 15 plates across 3 independent differentiation batches. *, *P* < 0.05; **, *P* < 0.01; ***, *P* < 0.001; ****, *P* < 0.0001.

As the application of VX-150 and SSCI-2 in both distal and middle compartments, that is, covering the entire length of the processes, effectively blocked the responses to electrical stimulation, we next investigated how the location of the application (i.e., the specific microfluidic compartment) of the blockers influenced their ability to reduce soma responses to stimulation of the distal processes. We tested the effectiveness of the blocker in either the distal (where the electrical stimulation was applied) or the hiPSC-SN soma (middle) compartments at DIV28-29 (Figure 5A). Application of SSCI-2 or VX-150 to hiPSC-SN at either the distal (mean fold change for SSCI-2 vs. IB: 0.11 [95% CI: −0.17 to −0.05], and VX-150 vs. IB: −0.05 [95% CI: −0.09 to 0.01], Figure 5B and C) or the soma (mean fold change for SSCI-2 vs. IB: −0.28 [95% CI: −0.34 to −0.22], and VX-150 vs. IB: −0.07 [95% CI: −0.01 to 0.02], Figure 5B and D) led to a significant reduction in the mean amplitude of the soma responses to the electrical stimulation of the distal compartment. We also found no significant difference in the proportion of cells showing a reduction in response amplitude of 40% or more when SSCI-2 was applied to either compartment (mean difference in proportions: 0.02 [95% CI: −0.14 to 0.15]; Figure 5E) However, a significantly higher proportion of neurons showed a reduction in response amplitude of 40% or more when VX-150 was applied to hiPSC-SN processes in the distal compartment compared with imaging buffer control (mean difference in proportions, VX-150 vs. IB: 0.10 [95% CI: 0.06-0.15]; Figure 5F). These data support a potential differential effect of the Nav1.8 blocker VX-150 depending on the site of application along the length of the hiPSC-SN processes.

## Discussion

In this study, we established microfluidic cultures of hiPSC-derived sensory neurons in monoculture and in co-culture with hiPSC-derived cortical neurons, providing a human relevant model for studying signaling in human sensory neuron processes. While previous studies have demonstrated the successful differentiation of iPSC-derived motor and cortical neurons in microfluidic systems,^35^^,^^36^ no prior work has examined hiPSC-derived human sensory neurons in this context until now. The compartmentalized design enabled targeted manipulation of distal and soma compartments and functional assessment of signaling in the microfluidic cultures. The Interpretation of these findings is based on prior demonstrations of effective fluidic isolation and spatially restricted stimulation in comparable microfluidic cultures.^10^^,^^11^^,^^26^ Although full maturation of iPSC-derived neurons remains a challenge,^37^ microfluidic co-culture systems may provide a framework for further improving the physiological relevance and utility of these models.

We demonstrated the successful differentiation and maturation of hiPSC-SN in microfluidic cultures both in mono- and co-culture configurations. We showed that stimulation of the hiPSC-SN distal processes lead to responses in the soma and confirmed the expression of Nav1.7 and Nav1.8 mRNA using qPCR and RNAscope in sensory neurons. Functional assays using Nav1.7-specific inhibitor SSCI-2 and Nav1.8-specific inhibitor VX-150 demonstrated significant reductions in neuronal soma responses to stimulation of distal processes, consistent with a functional contribution of these channels to signal propagation along these processes in microfluidic cultures. We did not characterize the nociceptor subtypes present in the microfluidic cultures. Accordingly, the pharmacological findings were interpreted in the context of a nociceptor-enriched sensory neuron population.

Previous studies have focused on the responses of the soma to stimulation in hiPSC-SNs.^38^ In this study, we demonstrate Nav1.7- and Nav1.8-dependent functional responses in hiPSC-SN processes. SSCI-2 and VX-150 are selective inhibitors of Nav1.7 and Nav1.8, respectively, and were used here to assess channel-dependent signaling in hiPSC-SN processes. Our results demonstrate the effectiveness of VX-150 and SSCI-2 in inhibiting signaling along human sensory neurons.

Here, we show that application of high concentrations of growth factors to the distal hiPSC-SN processes increased somal responses to stimulation of these processes, in a manner reminiscent of sensitization described in other systems.^10^^,^^39–41^ Although an effect on maturation-related properties cannot be excluded, our findings support the potential of using microfluidic cultures of hiPSC-derived sensory neurons to study mechanisms of growth factor-mediated modulation of responses in human nociceptive processes in vitro.

Consistent with previous findings in rodent sensory neurons,^42^^,^^43^ we observed function contribution of Nav1.7 to signal propagation along the hiPSC-SN processes and soma. In iPSC-derived nociceptors, Nav1.7 has been found at the soma, along axons, and enriched in terminal structures.^44^ Our compartmentalized microfluidic culture experiments revealed that Nav1.7 blocking efficiency varied depending on location of the application. In monocultures, SSCI-2 application in the middle compartment isolating the middle segments of the sensory processes significantly reduced the responses to electrical stimulation of the distal compartment. Previous studies have validated the fluidic isolation in microfluidic devices under similar experimental conditions suggesting minimal leakage of blocker between the compartments.^45^ In co-cultures, blocking Nav1.7 with SSCI-2 in either the soma or distal process compartments led to reduction in soma responses to stimulation of the processes, consistent with a functional contribution of Nav1.7 at multiple locations within the hiPSC-SN processes. In this context, the co-culture experiments were included as a development step for assessing sensory neuron function in a more complex human cellular model, although mature synaptic interaction between sensory and cortical neurons was not detected under the present conditions.

For Nav1.8, which is primarily expressed in sensory neuron soma and nerve terminals,^46^ VX-150 application also showed location-dependent effects. In monocultures, VX-150 significantly reduced responses when applied to the middle compartment. In co-cultures, VX-150 application to the distal compartment led to a significantly higher proportion of neurons with responses inhibited by more than 40%, compared to application to the middle compartment. Although this data does not directly establish the channel protein localization or subcellular distribution, it is consistent with a differential effect of VX-150 depending on the site of application along the sensory neuron processes.

We next investigated evidence of synapse formation between hiPSC-SNs and hiPSC-CNs. While previous studies have reported synaptic activity within monocultures of hiPSC-CNs ^47^ or in co-cultures with glial cells, particularly astrocytes,^48^ here we found no strong evidence of synaptic communication between 2 hiPSC-SNs and hiPSC-CNs. Only a single cell trace exhibited a response pattern suggestive of synaptic interaction, but this was insufficient to draw firm conclusions. Given that our experiments did not extend beyond DIV34, it is possible that the neurons were too immature for proper synapse formation. If neuronal maturity is a limiting factor, co-culturing with astrocytes could enhance synaptic development, as suggested by previous reports.^49^ Although the compartment-specific MAP2/peripherin staining patterns and functional responses support successful differentiation under the present conditions, the influence of co-culture media conditions on lineage stability was not directly tested in this study and remains an important area for future investigation. Alternatively, the absence of functional synapses between iPSC-derived sensory and cortical excitatory neuronal types could be an intrinsic feature of this model, suggesting that spinal cord, and not cortical neurons, are needed as partners for synapse formation.

In conclusion, we have established and validated a microfluidic culture model based on human iPSC-derived neurons to study human sensory neuron processes. We have provided evidence consistent with a functional contribution of Nav1.7 and Nav1.8 channels to signal propagation along these processes in microfluidic cultures. We also establish the potential of the platform for building more complex in vitro models of human pain system. Ultimately, our findings highlight the significant potential of using hiPSCs within microfluidic platforms to develop and test novel analgesic compounds in a physiologically relevant human context.

## Supplementary Material

szag065_Supplementary_Data

## Data Availability

All data reported in this manuscript have been included in the results or as Supplementary Material. All reasonable requests for data will be considered and fulfilled if possible.
