# Supplementary material for: Functional characterization of human iPSC–derived sensory neuron processes in microfluidic cultures
Source: Stem Cells Transl Med. 2026 Aug 8;15(8):szag065. doi: 10.1093/stcltm/szag065 (PMC13453313; doi:10.1093/stcltm/szag065)
Supplement: Supplementary file 1 [file szag065_supplementary_data.docx]

**Functional characterisation of human iPSC-derived sensory processes in microfluidic cultures.**

Laura Kleckner^1^, Omar Mossad^2^, Anil Kumar Kalia^2^, and Ramin Raouf ^1*^

1 – Wolfson Sensory, Pain and Regeneration Centre (SPaRC), Institute of Psychiatry, Psychology & Neuroscience, King’s College London, London, United Kingdom

2- Grünenthal GmbH, Aachen, Germany

***** Correspondence to Dr Ramin Raouf, Wolfson Sensory, Pain and Regeneration Centre (SPaRC), King’s College London, Guys’s Campus, London, SE1 1UL, United Kingdom (email: ramin.raouf@kcl.ac.uk)

**Supplementary Figures and Table**

**Figure S1**

**Figure S1: Brightfield Images of microfluidic co-cultures of hiPSC-SN and hiPSC-CN.** For brightfield imaging**,** cortical neural stem cells were maintained and differentiated in 24 well plates for 10 days and were then transferred to microfluidic devices containing hiPSC-SNs (see Methods). Images were acquired at 10x magnification from two different microfluidic cultures). **A and B)** Sensory processes in the distal compartment at DIV27. Neurite bundles growing out of the microfluidic microgrooves into the distal compartment are visible in **A** (at the top). **C and D**) hiPSC-SN in microfluidic culture. The extensive growth and arborisation of the processes within the soma compartment is clearly visible. **E and F)** hiPSC-CNs in microfluidic culture. Extensive branching of the processes from the hiPSC-CNs is visible, as well as processes fasciculate in bundles that exit from the microgrooves (originating from the iPSC-SN, not shown). Cells were fixed with PFA and visualised at DIV27. Scale bar 100µm

| **Table S1: Growth factors regimen** | | | |  |  | | |  | | |
| --- | --- | --- | --- | --- | --- | --- | --- | --- | --- | --- |
| **DIV 1 - 14** | | | | **DIV 14 – 29** | | | | | |  |
|  | iPSC-CN Soma | iPSC-SN Soma | Distal compartment | iPSC-CN Soma | | iPSC-SN Soma | Distal compartment (Low) | | Distal compartment (High) |  |
| NGF | 0 ng/mL | 25 ng/mL | 25 ng/mL | 100 ng/mL | | 25 ng/mL | 25 ng/mL | | 100 ng/mL |  |
| GDNF | 0 ng/mL | 25 ng/mL | 25 ng/mL | 0 ng/mL | | 25 ng/mL | 25 ng/mL | | 100 ng/mL |  |
| BDNF | 0 ng/mL | 10 ng/mL | 10 ng/mL | 20 ng/mL | | 10 ng/mL | 10 ng/mL | | 40 ng/mL |  |
| NT-3 | 0 ng/mL | 10 ng/mL | 10 ng/mL | 0 ng/mL | | 10 ng/mL | 10 ng/mL | | 40 ng/mL |  |

Summary of growth factor concentrations in hiPSC-derived sensory and cortical neuron media during differentiation. Concentrations were based on the manufacturer recommended differentiation protocol, with modifications from DIV14 onwards. Under the high growth factor condition, concentrations in the distal compartment were increased four-fold, and NGF was added to the hiPSC-CN compartment to promote outgrowth of hiPSC-SN processes into adjacent compartments. Under the low growth factor condition, concentrations were identical in the sensory soma and distal compartments.

**Figure S2**

**Figure S2: hiPSC-SN and hiPSC-CN soma responses to application of 40 mM KCl in co-culture.** Example traces of Cal520 fluorescence intensity in responses to bath application of 40mM KCl to either hiPSC-SN **(A)** or hiPSC-CN **(B)** compartments in a microfluidic co-culture at DIV29.

**Figure S3**

**Figure S3: Singular response of a cortical neuron to stimulation of sensory axons blocked by a cocktail of CNQX, APV, and Gabazine. A)** Schematic of the experimental setup. CNQX, APV and Gabazine were applied in the cortical compartment, while the distal compartment was electrically stimulated with a biphasic stimulus at 50Hz and 75µA. Ca^2+^ imaging recordings was performed in the hiPSC-CN compartment. **B)** Singular trace of Cal520 fluorescence intensity recorded from an iPSC-CN soma blocked with CNQX, APV, and Gabazine cocktail. Electrical stimulation of the sensory neuron processes in the distal compartment is marked with arrows. The responses from hiPSC-SN to stimulation of sensory neuron processes can clearly be observed in IB (Imaging buffer), but not after bath application of the blocker cocktail. The response partially recovered after the washout of the blockers (Wash IB), suggesting the existence of a synaptic contact between the hiPSC-CN and the hiPSC-SN processes at DIV34. No other such examples were observed.

**Figure S4**

**Figure S4: Concentration dependence of the inhibition of responses to stimulation of sensory neuron processes by SSCI-2 and VX-150. A)** Schematic of the experimental setup. The blockers were applied to both the distal and the sensory soma (middle) compartments. Ca2+ imaging recordings were performed in the sensory compartment, while the distal compartment was electrically stimulated. Both SSCI-2 and VX-150 experiments were performed at DIV27–29. **B)** Representative traces of Cal520 fluorescence intensity recorded from a hiPSC-SN soma before and during application of VX-150 (1 or 3 µM) (red) or SSCI-2 (50 or 100 nM) (green). The distal compartment was stimulated twice (marked with arrows) at 2 min intervals before each buffer exchange (IB, imaging buffer). **C)** Comparative effects of blocker concentration on the amplitude of somal responses to stimulation of the distal processes. Fold changes in the responses in the presence of the two concentrations of blocker or imaging buffer (vehicle control) are shown. There was no difference in the inhibition of the response between the two concentrations of SSCI-2 (mean difference, control vs. SSCI-2: 0.04 [95% CI -0.01 to 0.09]), while increasing the concentration of VX-150 from 1 µM to 3 µM led to a statistically significant reduction in the fold change in responses (mean difference, control vs. VX-150: 0.10 [95% CI 0.05 to 0.15]). One-way ANOVA, p-values: 0.59 and 0.004; N = 103, 331, and 280 for vehicle, SSCI-2, and VX-150, respectively. **D)** There was no change in the proportion of cells showing a 40% or greater reduction in response following application of the higher concentration of SSCI-2 (mean difference in proportions: 0.024 [95% CI -0.02095 to 0.06967]). Fisher’s exact test: N = 331, 331; p = 0.33. **E)** The proportion of cells exhibiting 40% or greater inhibition was significantly increased at the higher concentration of VX-150 (mean difference in proportions: 0.10 [95% CI 0.06 to 0.14]). Fisher’s exact test: N = 280, 280; p < 0.0001. Data were collected from two independent differentiation batches.

**Figure S5**

**Figure S5:** **Expression of SCN9A and SCN10A mRNA within hiPSC-CN soma in co-culture hiPSC-SN**. mRNA labelling of hiPSC-CN via RNAscope at DIV27 with SCN9A (yellow), DAPI (blue), β3-tubulin (white) and SCN10A (magenta) (A), SCN9A and β3-tubulin (B) and SCN10A with β3-tubulin (C). Low to sparse signal for SCN9A and sparser SCN10A mRNA signal in subset of the hiPSC-CN cell bodies is observed. Scale bar 20 μm
